# Hepatocyte Growth Factor Level in Cerebrospinal Fluid as an Additional Marker in Patient with Drug-Resistant Streptococcus Pneumoniae Meningitis Treated With Linezolid

**DOI:** 10.4137/ccrep.s759

**Published:** 2008-05-27

**Authors:** Hung-Chin Tsai, Yao-Shen Chen, Susan Shin-Jung Lee, Yu-Hung Lin, Shue-Ren Wann, Cheng Len Sy, Yung-Ching Liu

**Affiliations:** 1 Section of Infectious Diseases, Department of Medicine, Kaohsiung Veterans General Hospital, Kaohsiung, Taiwan, National Yang-Ming University, Taipei, Taiwan, Republic of China.; 2 Graduate Institute of Medicine, Kaohsiung Medical University, Kaohsiung, Taiwan, Republic of China.

**Keywords:** drug-resistance, hepatocyte growth factor, linezolid, meningitis, *Streptococcus pneumoniae*

## Abstract

Hepatocyte growth factor (HGF) is a multifunctional cytokine that has well-defined mitogenic, motogenic, and morphogenic functions on the epithelial cells. Strong increases of HGF concentrations in cerebrospinal fluid (CSF) are only present in patients with acute bacterial meningitis. We report a 15-year-old young man with drug resistant *Streptococcus pneumoniae* meningitis complicated with right 6th cranial nerve palsy. He presented with fever, headache and neck stiffness for 3 days and was treated with intravenous antimicrobial agents including linezolid and dexamethasone successfully. An association between CSF HGF concentrations and CSF proteins is observed (r = 0.897, p = 0.039. Pearson correlation test). This result showed that HGF level in CSF might act as an additional marker in patients with bacterial meningitis.

## Introduction

Hepatocyte growth factor (HGF) is a multifunctional cytokine that has well-defined mitogenic, motogenic and morphogenic functions on the epithelial cells.^1^,^2^ It has been proposed, from studies on the localization of the c-Met/HGF receptor in the brain and the interaction with HGF after brain injuries, that HGF plays an important role as a neurotrophic factor in the brain.^3^ An intrathecal production of HGF was found in acute/probable bacterial meningitis.^4^ However, the dynamic changes of HGF in CSF during the course of the disease in patient with bacterial meningitis is unknown. We present here a young man with drug-resistant *S. pneumoniae* meningitis who was followed up by serial CSF HGF levels.

## Case Report

A 15 year-old young man presented to our hospital with fever, neck stiffness and headache for 3 days. He had a history of right distal radius fracture 4 years ago and recovered completely. At presentation, physical examination revealed an acutely ill-looking, febrile patient with evident nuchal rigidity. Neurologic examination showed right 6th cranial nerve palsy. His blood pressure was 113/74 mmHg, pulse rate 88/min, and respiratory rate 18/min. Laboratory results showed a white cell count of 18.4 × 10^9^/l with granulocyte 81%, lymphocyte 12% and monocyte 6%. Platelet count was 513000/mm^3^. Lumbar puncture revealed an opening and closing pressure of 310 mmH2O, and 195 mmH2O, respectively. His CSF was turbid with a white cell count of 36/mm^3^ with 63% granulocyte, lymphocyte 30% and monocyte 7%. CSF protein level was 236 mg/dl and sugar 36 mg/dl (serum glucose 131 mg/dl). A gram stain of the spinal fluid showed numerous polymorphonuclear leukocytes and gram positive cocci in pairs and in chains. Since it was a holiday, the CSF was left at room temperature for 10 hours before processing. Culture of the CSF and blood yielded *Streptococcus pneumoniae* (MIC 2 ug/ml for penicillin, MIC 1.5 ug/ml for ceftriaxone by Phoenix 100, B.D. Biosciences, U.S.A.). He was treated with meropenem 2 g every eight hours with simultaneous intravenous dexamethasone and glycerol. Fever subsided 2 days later but recurred after 4 days of treatment. A repeat lumbar puncture showed a white cell count of 4850/mm^3^, with 99% granulocyte. CSF protein was 159 mg/dl and sugar 56 mg/dl. Blood culture again yielded *Streptococcus pneumoniae*. Echo-cardiogram showed absence of any intracardiac vegetation. Antibiotics was switched to vancomycin 1g every 12 hours and cefotaxime 2 g every 6 hours intravenously. His fever subsided but the headache persisted. On the 15th day of admission, rifampin at a dose of 600 mg every 12 hours was added due to recurrence of fever. No skin rash or eosinophilia was found. Another repeat lumbar puncture was done 2 days later showing a white cell count of 84/mm^3^, with CSF protein 68 mg/dl and sugar 49 mg/dl. Due to persistent fever and headache, all antimicrobial agents were discontinued and shifted to intravenous linezolid 600 mg every 12 hours. His fever subsided 4 days after. Brain magnetic resonance imaging showed presence of leptomeningeal enhancement. A follow up lumbar puncture done after 6 days of linezolid showed a white cell count of 80/mm^3^ with CSF protein level of 101mg/dl and sugar 43 mg/dl. Intravenous linezolid was given for 7 days and he was discharged under stable condition. He was followed up at out patient department for 1 year without any neurologic sequelae.

The CSF samples stored at −70 ºC were centrifuged at 3000 g for 15 min prior to analysis. Dynamic changes of CSF HGF concentrations was determined by ELISA, using a commercially available kit (Hu HGF Elisa Kit, BioSource Europe S. A, Belgium). The serial CSF parameters and levels of HGF are shown in Table 1. There was an association between HGF levels in CSF and CSF protein (r = 0.897, p = 0.039, Pearson correlation test).

## Discussion

The present study showed that changes in CSF HGF concentrations are correlated with levels of CSF protein. Linezolid may act as an alternative treatment for patients with drug-resistant *S. pneumoniae* meningitis. In a meningitis study done in 2004, Turkish researchers found that there were no significant correlations between HGF levels in CSF and other parameters of CSF, including cell count, total protein and glucose levels.^5^ Tsuboi et al. also found no relationship between HGF in CSF and CSF cells elements, protein, immunoglobulin index, or Q albumin. The CSF HGF value is highest in patients with acute disseminating encephalomyelitis.^6^ However, no patient with bacterial meningitis was included in their study. Our patient had dynamic CSF HGF levels that correlate with levels of CSF protein. An increase in CSF protein concentrations may be due to a decreasing CSF flow rate and appearance of plasma proteins in the CSF due to presumed or overt disruption of blood-CSF barrier.^7^,^8^ Hence this implies that the elevated CSF HGF concentrations in our patient probably caused in part by disruption of the blood brain barrier as well as intrathecal production of HGF.^4^ In a prospective observational study in France,^9^ researchers studied the effects of adjunctive corticosteroid on levels of vancomycin in CSF in patients with pneumococcal meningitis. Cefotaxime (200mg/kg/day), and high dose vancomycin (continuous infusion of 60 mg/kg/day after a loading dose of 15 mg/kg) were used and 43% (6/16) had intravenous rifampin added at a dose of 20 mg/kg/day as an adjunctive therapy. Totally, 14 patients were enrolled and the mean levels of vancomycin in the serum and CSF were 25.2 and 7.2 mg/L, respectively. Our patient used only the regular dosage of vancomycin (15 mg/kg/q12 h), thus a high CSF concentrations may not be reached. Linezolid is the first of a new class of antimicrobial agents, the oxazolidinones. It has excellent CSF penetration (70% of serum).^10^ Although it is not approved by U.S. Food and Drug Administration as a treatment option for drug-resistant *S. pneumoniae* meningitis, researchers conducting a study in neurosurgical patients found that the maximum and minimum measured concentrations of linezolid in the CSF were 10.8 g/ml and 6.1 g/ml.^10^ The penetration ratio of linezolid as represented by the ratio of the AUC for CSF to the AUC for serum was 0.66 and the mean elimination half-life of linezolid in CSF was 19.1 hours. The most important information from that study is that the high CSF concentrations of linezolid were derived from patients receiving neurosurgical procedures and only few of them have meningitis. Therefore, due to presence inflammation of the leptomeninges, our patient could have an even higher CSF concentration and this may be the reason for the success of the treatment. We switched the treatment to linezolid due to persistent fever and headache. However, the possibility of drug fever can not be excluded, as evidenced by the declining CSF protein, and cell count in the follow up lumbar puncture and fever subsided 4 days after the offending drugs were discontinued. Because of its favorable pharmacokinetic profile, linezolid has been increasingly administered for gram-positive cocci CNS infections.

In conclusion, we suggest that CSF HGF concentrations might provide additional information in patients with bacterial meningitis. Although further study is needed to verify our observation, we suggest that linezolid may act as an alternative treatment choice for patients with drug-resistant *S. pneumonaie* meningitis.

## Figures and Tables

**Table 1:** CSF parameters and levels of HGF in a 15 years-old patient with drug-resistant *S. pneumoniae* meningitis.

Day of admission	1	7	15	17	21
CSF white cell count (cell/mm^3^)	36^a^	4850	420	84	80
CSF protein (mg/dl)	236	159	122	68	101
CSF sugar (mg/dl)	36	56	72	49	43
CSF HGF (pg/ml)	6570	3886	251.2	1244	150.7

a The CSF was left in room temperature for 10 hours before processing because of the Chinese New Year holiday.
